# Triple-Targeted DNA Nanozyme Executes Disulfidptosis for Glioma Elimination

**DOI:** 10.7150/thno.133314

**Published:** 2026-03-30

**Authors:** Tiantian Wu, Le Li, Shun Zhang, Guannan Zhang, Chen Li, Jiaming Mei, Xiaoyuan Chen, Junjie Cheng

**Affiliations:** 1 NHC Key Laboratory of Tropical Disease Control, School of Life Sciences and Medical Technology, Hainan Academy of Medical Sciences, Hainan Medical University, Haikou, Hainan, 571199, China; 2 Department of Neurosurgery, The First Affiliated Hospital of USTC, Division of Life Sciences and Medicine, University of Science and Technology of China, Hefei, Anhui Province, PR China; Anhui Province Key Laboratory of Brain Function and Brain Disease, Hefei, Anhui Province, China.; 3 Shandong Provincial Key Laboratory of Precision Oncology, Shandong Cancer Hospital and Institute, Shandong First Medical University and Shandong Academy of Medical Sciences, Jinan 250117, China.; 4 Department of Nutrition and Food Hygiene, School of Public Health; Department of Radiology, Zhongda Hospital, Nurturing Center of Jiangsu Province for State Laboratory of AI Imaging & Interventional Radiology, School of Medicine; Southeast University, Nanjing, China Southeast University, Nanjing, 210009, China.

**Keywords:** nanomedicine, DNA nanotechnology, DNA biomaterials, nanozyme, tumor therapy

## Abstract

**Rationale:**

Targeting metabolic vulnerabilities, particularly mitochondrial dysfunction, has emerged as a promising therapeutic strategy for glioma. However, the precise induction of specific cell death pathways *via* non-genetic nanotherapeutics remains a significant challenge. Here, we report a triple-targeted DNA nanozyme designed to eliminate aggressive glioma by precisely inducing disulfidptosis.

**Methods:**

A programmable nanoplatform, termed TMGH@AD, was constructed *via* rolling circle amplification. This system integrates three specific targeting ligands to facilitate blood-brain barrier (BBB) penetration, tumor accumulation, and mitochondrial localization. It also incorporates a G4/Hemin DNAzyme with peroxidase-mimicking activity and the mitochondrial-disrupting agent Alexidine (AD). The therapeutic efficacy and mechanism of action were evaluated both in vitro and in vivo.

**Results:**

Following systemic administration, TMGH@AD achieved sequential delivery to brain tumors and accurate mitochondrial localization. Within the mitochondria, the DNAzyme catalyzed the in situ generation of hydroxyl radicals (·OH). The synergistic reactive oxygen species (ROS) burst derived from the G4/Hemin nanozyme and the released AD triggered PTPMT1 dysregulation, catastrophic oxidative stress, and lipid peroxidation. This cascade led to substantial NADPH depletion and intracellular disulfide accumulation, ultimately executing disulfidptosis as the dominant cell death pathway.

**Conclusions:**

This study presents a novel DNA nanozyme-based strategy that combines precise triple-targeted delivery with disulfidptosis activation. By overcoming delivery barriers and exploiting metabolic vulnerabilities, TMGH@AD offers a powerful therapeutic avenue for glioma eradication.

## Introduction

Cancer has been increasingly recognized as a disease characterized by profound metabolic reprogramming rather than simple uncontrolled proliferation.^1^-^3^ Mitochondria are at the center of this metabolic shift, serving not only as energy producers but also as dynamic regulators of bioenergetics, biosynthesis, redox homeostasis, and cell death. Furthermore, they are the primary regulators of redox homeostasis and the intrinsic pathway of apoptosis, making them key determinants of cell fate under stress conditions induced by therapy. It has been demonstrated that disturbance of mitochondrial homeostasis is tightly linked to the pathogenesis and development of cancer. In cancer cells, mitochondria are reprogrammed to support rapid proliferation by maintaining biosynthetic precursor generation and redox homeostasis, while modulating various cell death pathways.^4^-^7^ Consequently, mitochondrial dysfunction is no longer considered a mere byproduct of oncogenesis but is now understood as a critical driver of tumor initiation, progression, and metastasis. Increasing evidence suggests that mitochondria-targeted treatment exhibits higher therapeutic efficacy while reducing systemic toxicity. Such approaches maximize the therapeutic index, minimize drug dosage, overcome multidrug resistance, and hinder tumor recurrence and metastasis, as well as mitigate injuries to healthy tissues. 7-9 However, effective mitochondrial therapy remains difficult to achieve due to biological barriers such as the blood-brain barrier (BBB) and limited subcellular delivery. Moreover, existing treatments rarely induce irreversible mitochondrial-dependent cell death, such as disulfidptosis, which is driven by NADPH depletion and excessive disulfide bond formation.

In this context, nanotechnology offers a distinct advantage for the development of such precise interventions.^10^-^13^ By enabling the targeted delivery of agents that selectively exploit the vulnerabilities of cancer cell mitochondria, nanocarriers can improve therapeutic efficacy and controllability. Therefore, nanocarrier-mediated mitochondrial targeting represents a promising strategy to attack the metabolic and survival core of tumor cells. The convergence of DNA nanotechnology further expands this potential, providing a new class of biomaterials for biomedical applications.^14^-^19^ Among these, nanostructures fabricated *via* rolling circle amplification (RCA), known as DNA nanoflowers, have shown great promise in precision tumor therapy.^20^-^22^ The utility of these materials arises from three interconnected attributes: universality, programmability, and multifunctionality. The RCA process allows cost-effective synthesis of nanoflowers from almost any circular DNA template, while the template sequence can be rationally programmed to introduce functional domains such as aptamers, antisense oligonucleotides, or catalytic DNAzymes.^23^-^29^ This modularity transforms the nanoflower from passive carriers into active, multifunctional therapeutic systems capable of tumor recognition, drug release, and catalytic activity.^25^, ^29^-^31^ However, most existing DNA nanoplatforms focus on drug delivery or gene regulation, and few are designed to cross the BBB, achieve mitochondrial localization, and induce disulfidptosis.

**Scheme 1:**
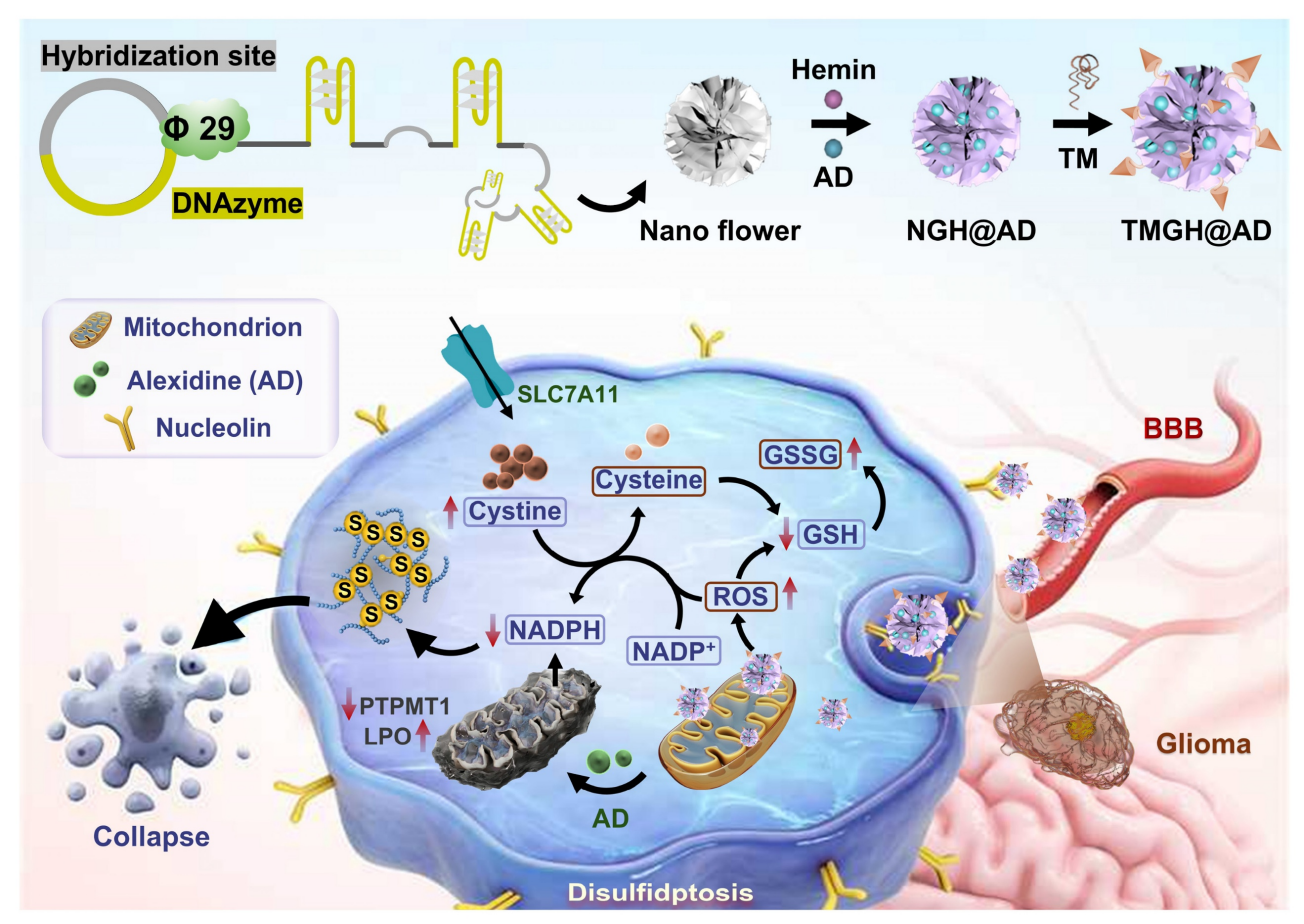
Schematic illustration of the construction and proposed treatment mechanism of the DNA nanozyme TMGH@AD (Triple mitochondrion-targeted G4/Hemin nanozyme loaded with Alexidine) in glioma treatment. TM: Triple mitochondrion-targeted DNA motif.

To address these challenges, a multifunctional triple-targeted DNA nanozyme (TMGH@AD) that integrates multiple targeting ligands and synergistic drug loading for the treatment of aggressive glioma (Scheme 1). This DNA nanozyme, namely TMGH@AD (Triple mitochondrion-targeted G4/Hemin nanozyme loaded with Alexidine), was constructed *via* RCA and subsequently co-assembled with a triple mitochondrion-targeted DNA motif through non-covalent interactions. The designed nanoplatform enables sequential delivery by crossing the blood-brain barrier, accumulating in glioma tissue, and localizing within mitochondria to disrupt mitochondrial homeostasis. Once internalized, the TMGH@AD specifically binds to mitochondria, catalyzing the generation of •OH radical and amplifying oxidative stress through the synergistic action of G4/Hemin and the released Alexidine. This process leads to lipid peroxidation, redox imbalance, and the catastrophic accumulation of intracellular disulfide bonds. These events ultimately trigger effective disulfidptosis as the predominant mode of cell death, providing a potent strategy to overcome delivery barriers and improve the therapeutic efficacy in glioma treatment.

## Results and Discussion

### Construction and characterization of the DNA nanozyme TMGH@AD

**Figure 1:**
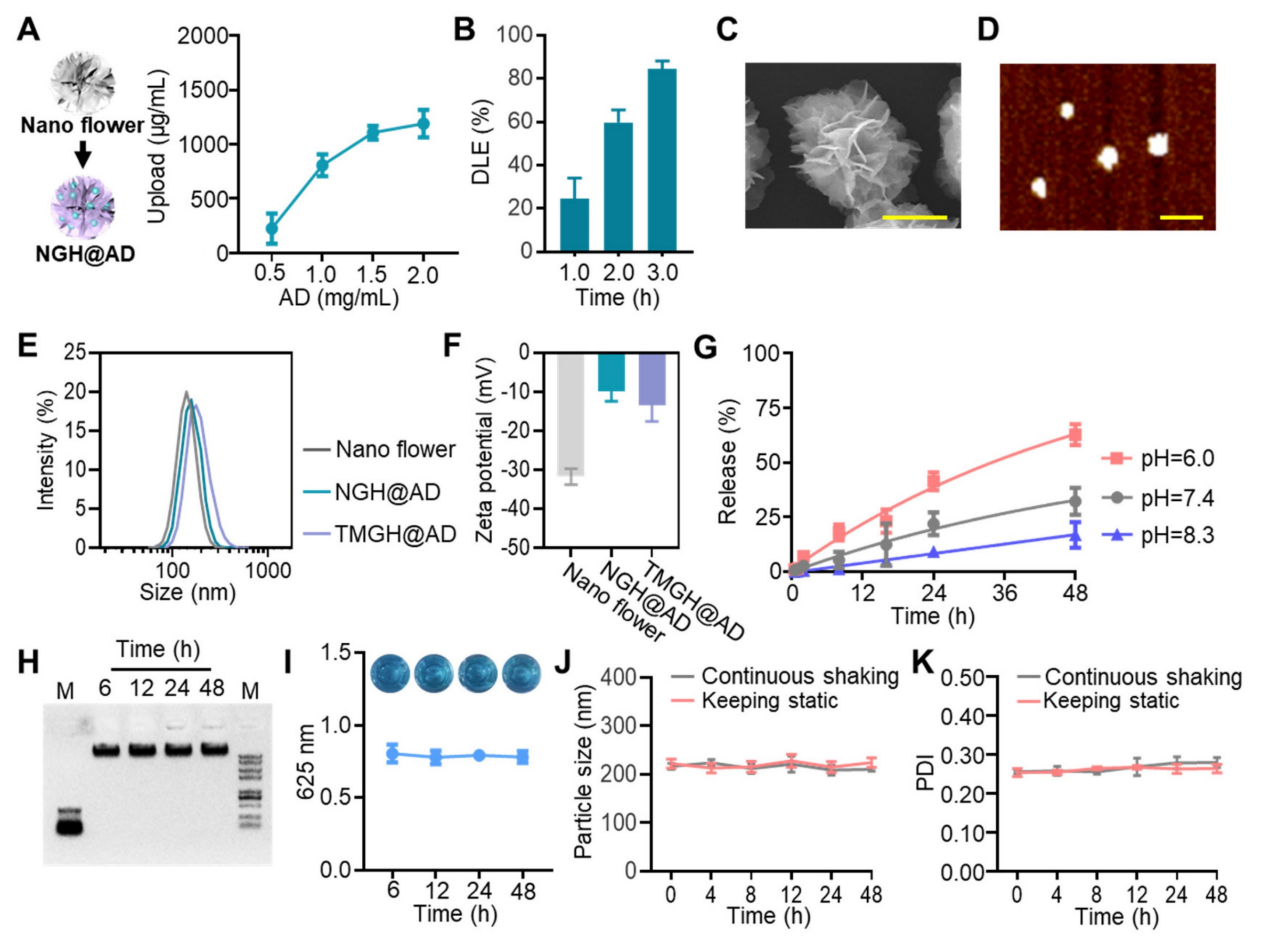
** Construction and characterization of the DNA nanozyme.** (A) Upload of AD in NGH@AD. (B) Drug loading efficacy (DLE) of AD measured by high-performance liquid chromatography. (C) Scanning electron microscopy (SEM) imaging of the TMGH@AD. Scale bar: 100 nm. (D) Atomic force microscope (AFM) imaging of the TMGH@AD under ScanAsyst-Air mode. Scale bar: 500 nm. (E) Hydrodynamic size analysis conducted by dynamic light scattering in PBS. (F) Zeta-potential of Nano flower, NGH@AD, and TMGH@AD. (G) Time-dependent AD release at different pH. (H) 0.8 % agarose gel electrophoresis of TMGH@AD in DMEM with 10% FBS. (I) TMB assay of TMGH@AD after different culture times. TMB oxidation was measured by recording absorbance at 652 nm. The photographs of the corresponding solutions are also shown as insets. Particle size (J) and Polydispersity (K) of TMGH@AD cultured under continuous shaking or static conditions.

The unique advantages of DNA nanotechnology make it a powerful platform for constructing nanoscale drug delivery vehicles for precise disease intervention.^32^-^34^ The RCA-based nanostructure was fabricated by initially generating a circular DNA template, which incorporated a hybridization site and a G4/Hemin DNAzyme motif. The fabrication was achieved through the annealing of a predesigned linear DNA strand with a primer, followed by enzymatic ligation using T4 DNA ligase. Details regarding the schematics and sequences are provided in Figure S1 and Table S1 in Supporting Information. The RCA employed in our study is pivotal for enhancing the peroxidase-mimetic activity of the G4/Hemin complex, which is crucial for the generation of highly reactive hydroxyl radicals (•OH) as toxic agents. RCA creates a multivalent nanoplatform that leads to an extremely high local concentration of G4/Hemin catalytic centers, which significantly amplifies the overall catalytic turnover rate through a proximity effect. The RCA-generated Nano flower with long, repeated ssDNA sequences effectively interacted with Hemin and Alexidine (AD) for drug loading and served as “scaffolds” that offer multiple predesigned hybridization sites for further modification. Unlike Hemin, which specifically binds to G4 sequence, AD was loaded into the Nano flower through electrostatic adsorption. To optimize the drug loading condition, we used high-performance liquid chromatography (HPLC) to analyze the AD loading and release performance (Figures 1A-B). The upload AD in DNA nanoflower increased with the initial adding amount but plateaued at 2 mg. A relatively high drug loading efficacy (DLE, 84.3 ± 2.4 %) of AD was achieved through 3-hour incubation, owing to efficient electrostatic adsorption between AD and DNA strand. As the targeting ligand for hybridization to the drug-loaded Nano flower (Nontargeted G4/Hemin nanozyme loaded with Alexidine, NGH@AD), the triple-targeted DNA device was created based on our previous research (Figure S2).^35^, ^36^ The self-assembly Y shape structure of the DNA device comprised with 2 anti-TfR aptamer GS24 and 3 Nucleolin-targeted aptamer AS1411 at specific site for blood-brain barrier (BBB) crossing, spheroid penetration, and enhanced cellular uptake. Moreover, 3 copies of mitochondrial targeting modification were introduced for further subcellular localization of the DNA nanozyme to the mitochondrion. The extended capture strand of the fabricated DNA device TM subsequently hybridized with the hybridization site of NGH@AD. After the final hybridization, the multifunctional DNA nanozyme (TMGH@AD, Triple mitochondrion-targeted G4/Hemin nanozyme loaded with Alexidine) was synthesized and characterized as a drug delivery system for glioma therapy.

As shown in Figures 1C-D and S3-4, the clear flower-like morphology was imaged by scanning electron microscopy (SEM) and atomic force microscopy (AFM), indicating successful fabrication and monodisperse morphology of the TMGH@AD. The hydrodynamic diameter measured by dynamic light scattering (DLS) showed that the TMGH@AD (194.3 ± 11.4 nm) was slightly larger in size than the nontargeted NGH@AD (183.9 ± 14.1 nm) owing to the successful targeted modification (Figure 1E). As shown in Figure 1F, the surface charge properties evaluated by measuring the zeta potential also showed progressive change from AD loading (positive charge) to targeted modification (negative charge), further confirming the successful construction of the TMGH@AD. Next, the release profile of AD from TMGH@AD was assessed under different pH conditions, demonstrating a clear time-dependent release pattern (Figure 1G), which is desirable for targeted drug delivery in cancer treatment. The stability of TMGH@AD under neutral physiological conditions was verified in DMEM with 10% FBS. After 48 h of incubation, negligible aggregation, degradation, or catalytic activity loss was detected (Figures 1H-I and S5-6), indicating excellent biostability. Additionally, the triple-targeted DNA nanozyme maintained excellent integrity and dispersibility under both static and continuous shaking conditions. In summary, the TMGH@AD possesses the potential to serve as a targeted agent for sequential drug delivery.

### Verification of the sequential delivery of the TMGH@AD

**Figure 2:**
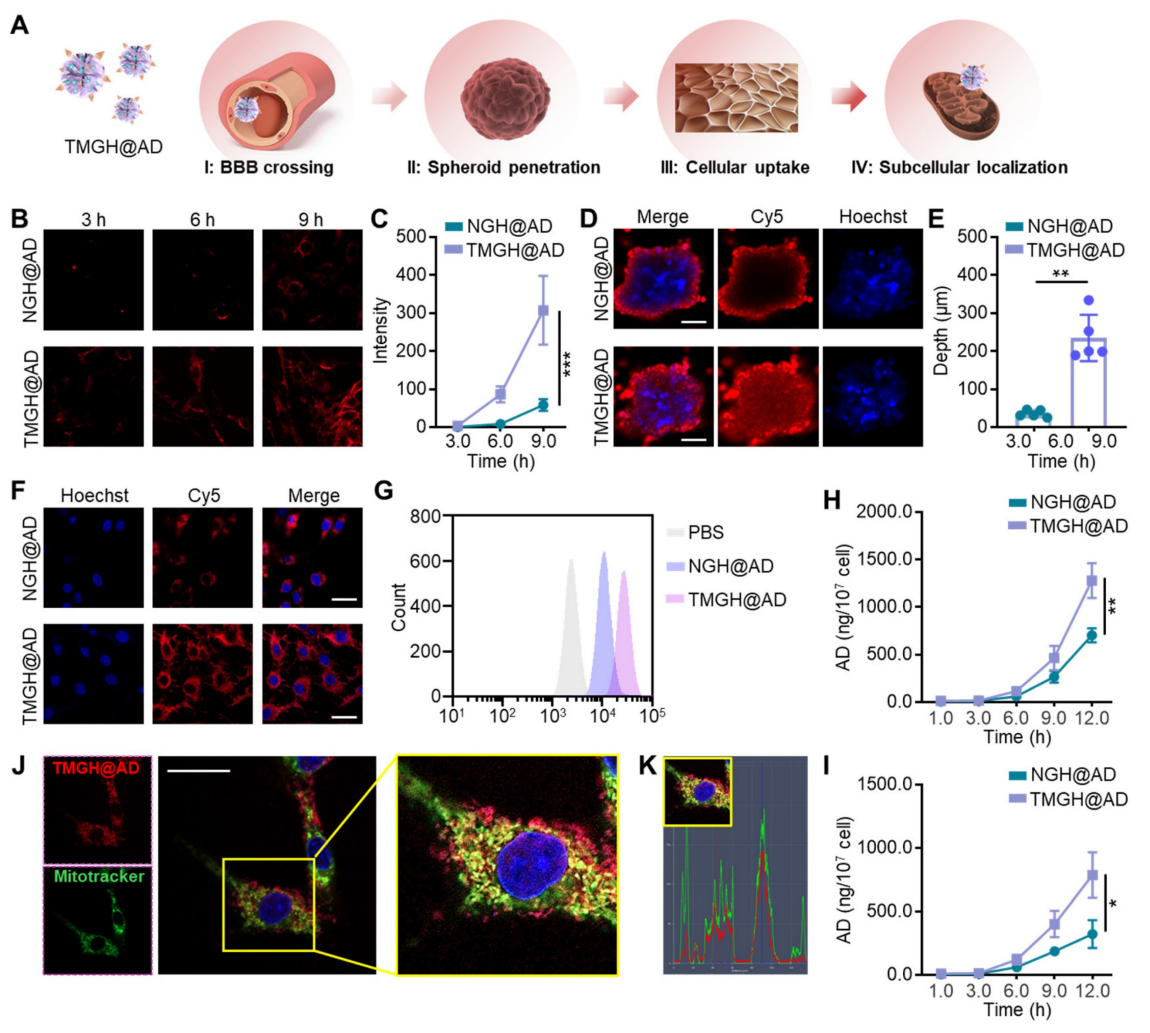
** Targeted delivery performance of the TMGH@AD.** (A) Illustration of the sequential delivery in blood-brain barrier (BBB)crossing, spheroid penetration, cellular uptake, and subcellular localization. (B-C) BBB crossing effect measured by *in vitro* BBB model. Cy5-labeled NGH@AD or Cy5-labeled TMGH@AD (1 µg/mL) was cultured with bEnd.3 model for 3 h, 6 h, and 9 h, and imaging by fluorescence microscope. (D-E) Penetration of the DNA nanozyme in GL261 spheroids. Scale bar: 500 µm. (F) Representative CLSM images of GL261 cells cultured with Cy5-labeled NGH@AD or TMGH@AD (1 µg/mL). Scale bar: 100 µm. (G) Flow cytometry analysis of the cellular uptake. AD accumulation in NGH@AD or TMGH@AD-treated GL261 cells (H) or spheroids (I) was measured by HPLC. (J) Subcellular location of NGH@AD. Red fluorescence represents Cy5-labeled TMGH@AD. Green fluorescence represents mitochondria stained with MitoTracker. (K) Co-localization of the TMGH@AD (Red) and mitochondrion (Green). (n=3). Statistical significance was calculated by one-way ANOVA with the Tukey post hoc test (* p < 0.05, ** p < 0.01, *** p < 0.001).

Effective nanocarrier-based drug delivery for glioblastoma is critically dependent on traversing the BBB, a formidable obstacle that blocks most chemotherapeutics.^37^ By engineering nanocarriers with BBB-crossing ligands to facilitate receptor-mediated transcytosis and subsequently combining with tumor cell targeting moieties, drug delivery efficiency can be significantly enhanced, enabling effective drug concentrations within the tumor.^37^, ^38^ In this study, the DNA nanozyme was equipped with anti-TfR aptamer GS24^39^ for BBB crossing Nucleolin-targeted aptamer AS1411^18^, ^40^ for tumor cell targeting, and triphenylphosphine for mitochondrion location. As hypothesized in Figure 2A, the TMGH@AD that guided by the modified targeting ligand, crosses BBB to reach the glioma site. It then penetrates the glioma, is efficiently internalized by the cells, and subsequently binds to mitochondria to exert its therapeutic effect. In the *in vitro* BBB model, the TMGH@AD exhibited a significant enhancement in crossing the cell layer than NGH@AD in a time-dependent way (Figures 2B-C). The fluorescence microscopy images clearly show that TMGH@AD is able to cross the BBB within 3 hours, with increasing accumulation observed at 6 or 9 h post-incubation. Next, we constructed 3D cell spheroids to evaluate the penetration effect. As shown in Figures 2D-E, TMGH@AD was successfully able to enter the GL261 spheroids with an average depth of 235.0 µm, whereas NGH@AD failed to reach the depths of the spheroids. In cell layer, a remarkable enhancement in cellular internalization was achieved with TMGH@AD compared to NGH@AD (Figure 2F). This enhanced uptake after 3 hours of treatment was also quantitatively confirmed by flow cytometry analysis (Figures 2G and S7). In AD accumulation analysis, the treatment of TMGH@AD achieved 1280.3 ng AD/10^7^ in cells and 786.7 ng AD/10^7^ in spheroids, while NGH@AD treatment was reduced by half (Figures 2H-I). Next, to analyze the subcellular localization of the TMGH@AD, the cells were stained with MitoTracker and the colocalization index. The CLSM images demonstrate the successful mitochondrial localization, as evidenced by the co-localization of Cy5 fluorescence (TMGH@AD) and MitoTracker green fluorescence (mitochondria) as shown in Figures 2J-K. Moreover, fluorescence imaging and HPLC quantification of AD of mitochondria isolated from DNA nanozyme-treated GL261 cells demonstrate that the TMGH@AD effectively bound to mitochondria following targeted modification (Figures S8-9).

### *In vitro* cytotoxicity, mitochondrion disruption, and disulfidptosis

**Figure 3:**
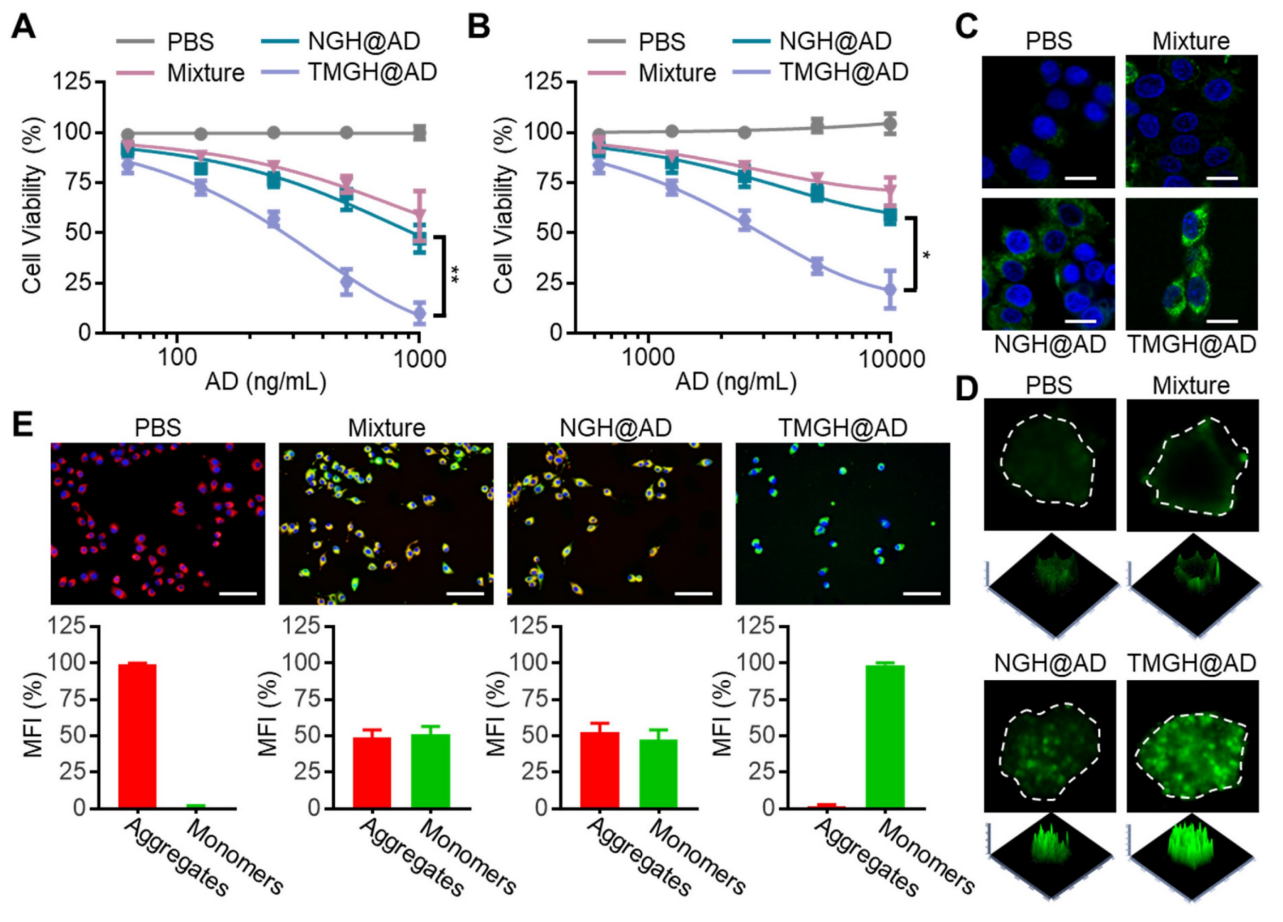
***In vitro* tumor inhibition effect of the DNA Nanozyme.** (A) Cell *via*bility analysis of GL261 cells treated with PBS, Mixture (Nano flower + TM + Hemin + AD), NGH@AD, or TMGH@AD. The drug concentration was based on AD (0 to 1000 ng/mL). (B) Cell *via*bility analysis of GL261 spheroids. The drug concentration was based on AD (0 to 10000 ng/mL). The representative CLSM images of the ROS production in GL261 cells (C) and spheroids (D). (E) JC-1 staining and analysis of GL261 cells with different treatments. Red fluorescence represents the aggregation signal, and green fluorescence represents monomer signal. The drug concentration was based on 1 µg/mL AD. Scale bar: 200 µm. (n=3). Statistical significance was calculated by one-way ANOVA with the Tukey post hoc test (* p < 0.05, ** p < 0.01).

To evaluate the *in vitro* anti-glioma efficacy of the DNA nanozyme, we compared the biological effects of AD, Nano flower, free components (Mixture: Nano flower + TM + Hemin + AD), NGH@AD, and TMGH@AD on GL261 cells and spheroids based on the same concentration of AD. The loaded AD in NGH@AD and TMGH@AD was quantified by HPLC through measuring the uploaded AD and the nuclease digestion product (standard de*via*tion < 5%). As shown in Figures 3A and S10, the results of CCK8 assays revealed a dose-dependent cytotoxicity. Notably, TMGH@AD exhibited the most potent inhibitory effect, significantly outperforming the non-targeted NGH@AD and the simple Mixture group (simple mixture of Nano flower + TM + Hemin + AD) across all tested concentrations. This highlights the critical role of the integrated DNA structure and active targeting in enhancing therapeutic efficacy. To better mimic the complex tumor microenvironment, we extended the investigation to GL261 spheroids. Consistent with the cell results, TMGH@AD induced the most substantial reduction in spheroid *via*bility, confirming its superior penetration and killing efficiency in a more physiologically relevant model (Figures 3B and S11). The underlying mechanism of this potent cytotoxicity was attributed to the robust generation of reactive oxygen species (ROS). The G-quadruplex/hemin (G4/Hemin) complex in DNA nanozyme functions as a peroxidase-mimicking nanozyme, effectively generating highly reactive and cytotoxic hydroxyl radicals (•OH) and other ROS.^41^, ^42^ As visualized by CLSM, both GL261 cells and spheroids treated with TMGH@AD displayed the strongest fluorescence signal generated by DCFH-DA treatment, indicating significantly higher intracellular ROS levels compared to all other treatment groups (Figures 3C-D and S12-13). Furthermore, the impact on mitochondrial function was investigated using JC-1 staining. As shown in Figures 3E and S14, TMGH@AD treatment caused a marked decrease in the red/green fluorescence ratio of JC-1. This shift from the aggregated (red) to the monomeric (green) state signifies a loss of mitochondrial membrane potential (MMP), confirming that compared to non-targeted NGH@AD, the specific subcellular location of TMGH@AD effectively induces mitochondrial damage.

**Figure 4:**
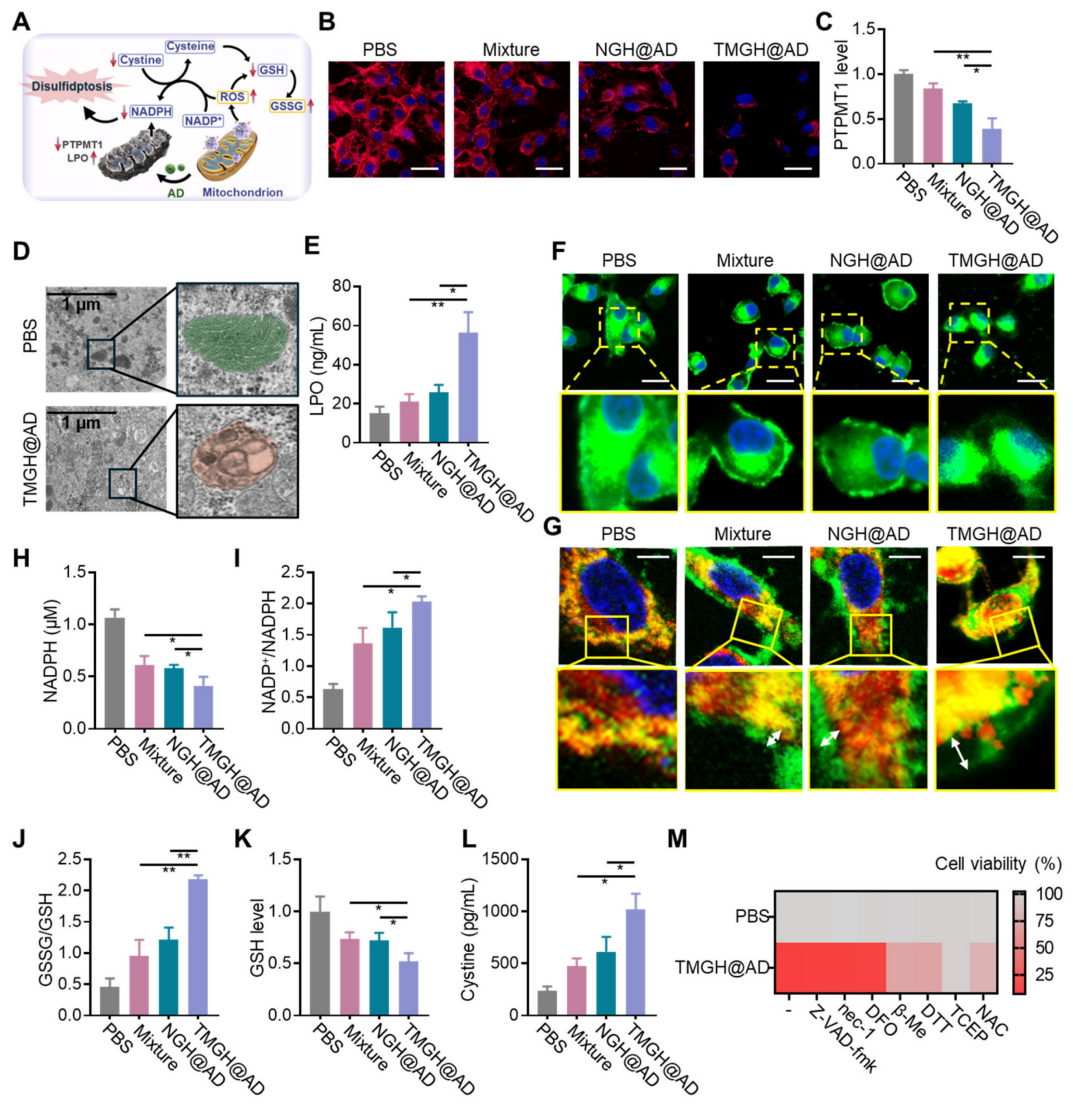
*** In vitro* mitochondrion disturbance and disulfidptosis induction by TMGH@AD.** (A) Illustration of the proposed mechanism of TMGH@AD in glioma treatment. (B) Immunofluorescence analysis of PTPMT1. Scale bar: 50 µm. (C) Relative expression level of PTPMT1 in GL261 cells. (D) TEM imaging of GL261 cells. (E) LPO level in GL261 cells. (F) Representative CLSM images of F-actin in GL261 cells. Scale bar: 50 µm. (G) Representative CLSM images of changes of F-actin in GL261 cells. Red fluorescence represents F-actin. Green fluorescence represents membrane. Scale bar: 5 µm. (H) Intracellular NADPH concentration, (I) NADP^+^/NADPH ratio, (J) GSSG/GSH, (K) GSH concentration, and (L) Cystine concentration after different treatments. (M) Heat map of relative cell *via*bility under different treatments. Cells were treated with PBS, Mixture (Nano flower + TM + Hemin + AD), NGH@AD, or TMGH@AD for 24 h. The drug concentration was based on 1 µg/mL AD. (n = 3). Statistical significance was calculated by one-way ANOVA with the Tukey post hoc test (* p < 0.05, ** p < 0.01).

Based on the above observations, we proposed a mechanistic model (Figure 4A), where the combination of* in situ* ROS generation and drug intervention induces mitochondrion homeostasis, leading to redox homeostasis, NADPH depletion, and finally causing disulfidptosis. As mentioned above, we have confirmed that the loss of MMP after the treatment of the DNA nanozyme. In this regard, PTPMT1 maintains MMP by regulating cardiolipin synthesis. Inhibition of PTPMT1 disrupts cardiolipin production, leading to instability of the electron transport chain, increased proton leak, and subsequent dissipation of MMP.^43^, ^44^ As shown in Figures 4B-C and S15, immunofluorescence analysis and quantification reveal that TMGH@AD treatment significantly downregulates the expression of PTPMT1 in GL261 cells compared to all other groups. The profound mitochondrial damage is further corroborated by Transmission electron microscopy (TEM) images, which display severe morphological disruptions, including cristae disappearance and vacuolization, in TMGH@AD-treated cells (Figure 4D). These results suggest the induction of mitochondrial permeability transition. Beyond structural damage, as shown in Figures 4E and S16, elevated levels of lipid peroxides (LPO) and their key byproduct malondialdehyde (MDA) are potent indicators of severe oxidative damage, indicating mitochondria-targeted interference conducted by TMGH@AD triggered irreversible damage in cells and activated cell death pathways related to oxidative stress. Subsequently, F-actin imaging was conducted to confirm that the DNA nanozyme treatment drastically impacts the cellular redox homeostasis and actin cytoskeleton. Figures 4F and S17 show that TMGH@AD causes a marked collapse of the F-actin cytoskeleton, a hallmark of disulfidptosis. The co-localization imaging analysis of CellMask and F-actin revealed a trend of separation between the red (membrane) and green (F-actin) fluorescence signals, indicating abnormal intermolecular disulfide bonds in actin (Figure 4G). This cytoskeletal collapse is driven by the aberrant formation of disulfide bonds between actin filaments. To confirm this, the study measured key redox-related metabolites. As shown in Figures 4H-K, TMGH@AD treatment leads to a significant depletion of intracellular NADPH and GSH, accompanied by a dramatic increase in the NADP⁺/NADPH and GSSG/GSH ratios. This depletion of reducing equivalents impairs the cell's ability to maintain cysteine in its reduced form, leading to cystine accumulation (Figure 4L). The resulting oxidative environment promotes the formation of lethal disulfide bonds, ultimately culminating in disulfidptosis.^45^-^47^ To further corroborate the mechanism of disulfidptosis, Different types of cell death inhibitors (Z-VAD-fmk for apoptosis inhibition; nec-1 for necrosis inhibition; DFO for ferroptosis inhibition) were introduced on the cells treated with TMGH@AD. After the treatment of TMGH@AD companied with 25 nM of Z-VAD-fmk, nec-1, or DFO, the cytotoxicity induced by the DNA nanozyme cannot be eliminated by inhibitors of other types of programmed cell death (Figures 4M and S18). Meanwhile, the cytotoxicity induced by TMGH@AD can be eliminated by reducing agent dithiothreitol (DTT), β-mercaptoethanol (β-Me), tris-(2-carboxyethyl)-Phosphine (TCEP), or N-acetylcysteine (NAC) in a dose-dependent way (Figure S19). These provide compelling evidence that the therapeutic effect of our DNA nanozyme is indeed based on the induction of disulfidptosis.

### *In vivo* anti-glioma efficacy

**Figure 5:**
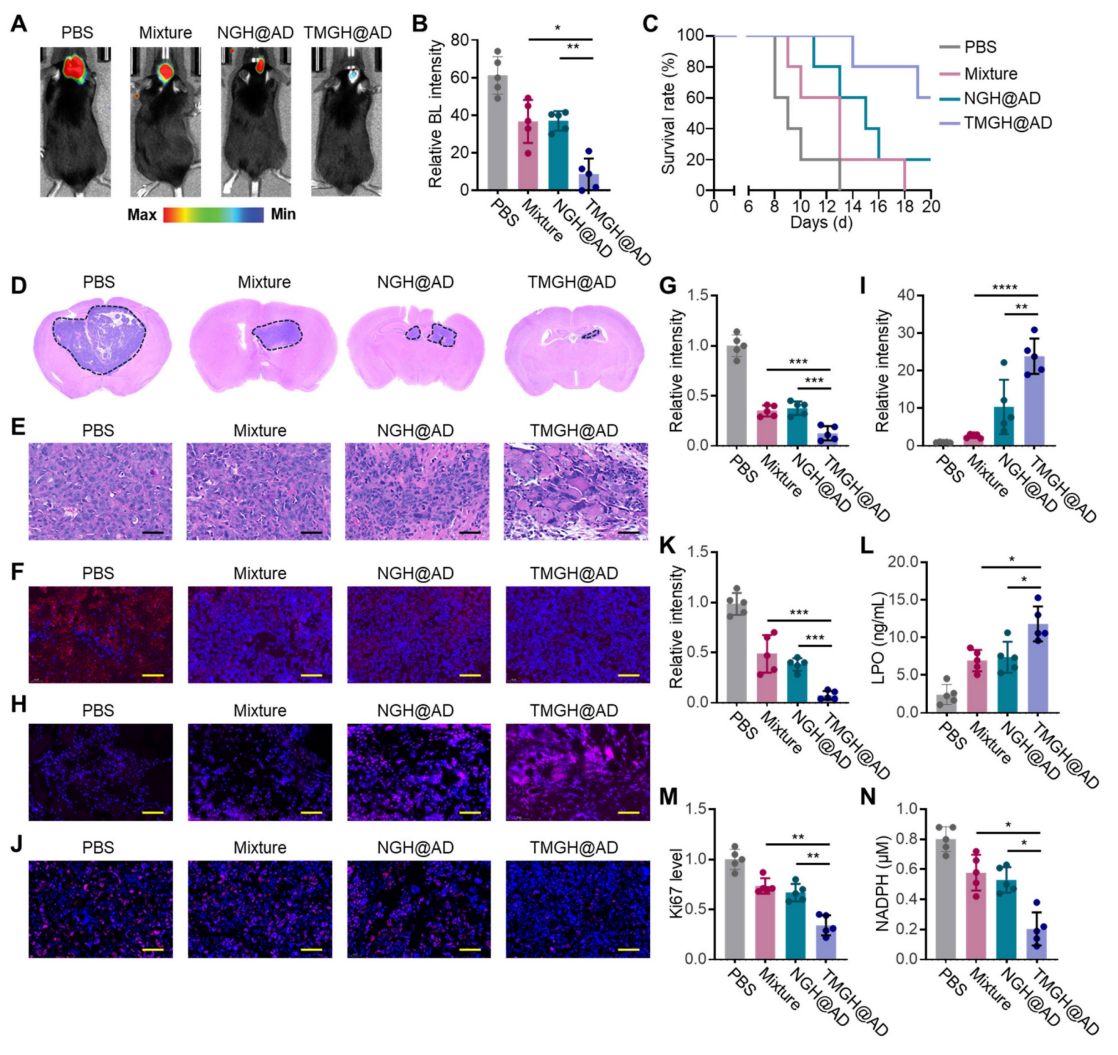
***In vivo* therapeutic effect of TMGH@AD.** (A-B) Bioluminescence (BL) imaging in brain at 7 Day of the treatment and corresponding quantification of the BL signal. The mice were intravenously (*i.v.*) injected with PBS, Mixture (Nano flower + TM + Hemin + AD), NGH@AD, or TMGH@AD with a dose of 2 mg AD/kg body weight at the first day of the treatment. (C) Survival curve of different treatments. (D-E) H&E staining of the brain collected from different groups. Scale bar: 40 μm. (F-G) Immunofluorescence staining and quantification of PTPMT1 in glioma. Scale bar: 40 μm. (H-I) ROS imaging and quantification. (J-K) Immunofluorescence staining and quantification of Ki67 in glioma. Scale bar: 40 μm. (L) LPO, (M) Ki67, and (N) NADPH level in the treated glioma tissue measured by ELISA. (n=5). Statistical significance was calculated by one-way ANOVA with the Tukey post hoc test (* p < 0.05, ** p < 0.01, *** p < 0.001, **** p < 0.0001).

The *in vivo* anti-glioma efficacy of the DNA nanozyme was evaluated in glioma model using C57 mice. The mice were randomized and treated with PBS, Mixture (Nano flower + TM + Hemin + AD), NGH@AD, or TMGH@AD *via* intravenous injection with a dose of 2 mg AD/kg body weight. The tumors in PBS exhibited rapid growth in 7 Day, in contrast, mice treated with TMGH@AD exhibited a dramatic reduction in Bioluminescence (BL) signal, as shown in Figures 5A-B, indicating a powerful inhibition of glioma growth. This superior therapeutic outcome directly translated to a significant survival benefit, as the survival curve reveals that TMGH@AD-treated mice had a markedly prolonged median survival time compared to all other treatment groups (Figure 5C). Histological and immunofluorescence analyses of harvested brain tissues were performed to corroborate the imaging data and elucidate the underlying mechanisms. Results of H&E staining confirmed that TMGH@AD treatment led to extensive tumor cell death and tissue necrosis, while tumors from control groups showed dense, *via*ble cell populations (Figures 5D-E). TUNEL staining further demonstrated that treatment with the TMGH@AD induced significant tumor cell apoptosis compared to non-targeted NGH@AD (Figures S20-21). Collectively, these *in vivo* results robustly demonstrate that TMGH@AD is a highly effective therapeutic agent for glioma, capable of suppressing tumor growth, extending survival, and inducing disulfidptosis through ROS-mediated mitochondrial damage and redox collapse. To confirm the induction of disulfidptosis *in vivo*, immunofluorescence analysis of the PTPMT1 was conducted. Results in Figures 5F and G demonstrate a significant downregulation of PTPMT1 in the TMGH@AD group, consistent with the *in vitro* findings of severe mitochondrial disturbance. The therapeutic effect was further attributed to the robust generation of ROS within the glioma microenvironment. ROS imaging and quantification revealed that TMGH@AD treatment induced the highest levels of intracellular ROS, far exceeding those in other groups (Figures 5H-I). This intense oxidative stress effectively suppressed tumor cell proliferation, as evidenced by immunofluorescence staining for the proliferation marker Ki67. As shown in Figures 5J and K, a marked decrease in the percentage of Ki67-positive cells in the TMGH@AD group confirmed the potent anti-proliferative activity. Quantitative ELISA measurements from glioma tissue lysates provided biochemical validation of the observed effects. As shown in Figures 5L-N, the TMGH@AD-treated tumors exhibited significantly elevated levels of LPO and depleted concentrations of NADPH, confirming the induction of the collapse of the cellular antioxidant system and disulfidptosis. In addition, the DNA nanozyme demonstrated a favorable biosafety profile, which was substantiated by the absence of significant weight loss in mice and by the results of blood analysis showing non-elevated inflammatory cytokine levels following its administration (Figures S22-23). Collectively, these *in vivo* results robustly demonstrate that TMGH@AD is a highly effective therapeutic agent for glioma, capable of suppressing tumor growth, extending survival, and inducing effective disulfidptosis through mitochondrial targeting interference.

## Conclusions

In summary, to address the challenge of treating highly aggressive and invasive gliomas, we have successfully engineered a multifunctional DNA nanozyme, namely TMGH@AD, designed to potently disrupt mitochondrial homeostasis. This nanoplatform, constructed *via* rolling circle amplification and subsequent co-assembly of DNA device, features triple targeting ligands and synergistic drug loading to achieve precise therapeutic delivery. The TMGH@AD nanozyme effectively navigates biological barriers to achieve sequential delivery to the brain tumor and subsequent subcellular localization within the mitochondria. Once localized, the TMGH@AD catalyzes the generation of powerful •OH radicals and, in synergy with the released AD, induces catastrophic oxidative stress and the accumulation of intracellular disulfide bonds. This cascade of events culminates in the effective induction of disulfidptosis as the predominant cell death modality. This innovative strategy, which overcomes critical impediments to drug delivery and enhances antitumor efficacy, presents a promising and powerful therapeutic avenue for the treatment of glioma.

## Supplementary Material

Supplementary figures and table.

## Data Availability

The data that support the findings of this study are available from the corresponding author upon reasonable request.
