# Supplementary material for: Triple-Targeted DNA Nanozyme Executes Disulfidptosis for Glioma Elimination
Source: Theranostics. 2026 Mar 30;16(10):5759–69. doi: 10.7150/thno.133314 (PMC13081161; doi:10.7150/thno.133314)
Supplement: Supplementary file 1 — Supplementary figures and table. [file thnov16p5759s1.pdf]

TABLE OF CONTENTS

Materials and cells S2

Experiment section S3-7

Supporting figures

Figure S1 .... S8
Figure S2 .... S9
Figure S3 .... S10
Figure S4 .... S11
Figure S5 .... S12
Figure S6 .... S13
Figure S7 .... S14
Figure S8 .... S15
Figure S9 .... S16
Figure S10 .... S17
Figure S11 .... S18
Figure S12 .... S19
Figure S13 .... S20
Figure S14 .... S21
Figure S15 .... S22
Figure S16 .... S23
Figure S17 .... S24
Figure S18 .... S25
Figure S19 .... S26
Figure S20 .... S27
Figure S21 .... S28
Figure S22 .... S29
Figure S23 .... S30

Supporting tables

Table S1 .... S31

References S32

1. Materials and cells

Chemicals and solvents were obtained commercially and used without further purification. Millipore water was used to prepare all aqueous solutions. All oligonucleotides and triphenylphosphine-modified DNA used in this study were purchased from Huzhou Hippo Biotechnology Co., Ltd.. 2′,7′-dichlorofluorescin diacetate (DCFH-DA), Trifluoroacetic acid, dithiothreitol, Hemin, N-acetylcysteine (NAC), β-mercaptoethanol (β-Me), TCEP and 3,3′,5,5′-tetramethylbenzidine sulfate (TMB) were purchased from Aladdin (Shanghai). JC-1 kit , Z-VAD-fmk, nec-1, and DFO was purchased from Merck & Co, Inc. DMEM, trypsin, and fetal bovine serum (FBS) were purchased from Gibco™. Antibodies and fluorescent probe-labeled secondary antibody were purchased from Abcam (Cambridge, MA) and Detaibio Co.,Ltd. Splint R ligase and Phi29 ligase were purchased from New England BioLabs. Cell culture dishes/plates, round coverslips, 20-mmglass-bottom dishes, and centrifuge tubes were obtained from NEST Biotechnology Co. Ltd. (Wuxi, China). YeaRed Nucleic Acid Gel Stain (cat#10202ES76), Hifair® III One Step RT-qPCR SYBR Green Kit (cat#11143ES70), GMyc-PCR Mycoplasma Test Kit (cat#40601ES20), and Necrostatin-1(Nec-1, cat#53771ES50)were purchased from Yeasen, Shanghai, China. Hochst33258 (C0020) and cell counting kit 8 (CCK-8) were purchased from Beijing Solarbio Science & Technology Co.,Ltd.. BioSci® New Flash Protein anyKD PAGE (8012011) was purchased from Dakewei Biotechnology Co., Ltd.. SDS-PAGE Gel Rapid Preparation Kit (KGP103) and BCA Protein Quantitation Assay Kit (KGP902) were purchased from KeyGEN BioTECH.

All cell lines used in this study were purchased from the American Type Culture Collection (ATCC). The cells were maintained in DMEM supplemented with 10% FBS and penicillin/streptomycin in a humidified atmosphere containing 5% CO_2 at 37 °C.

2. Experiment section

Design of the DNA template

The linear template containing the hybridization site and DNAzyme sequence (Table S1) were synthesized by solid-phase synthesis with phosphate at the 5' site. The primers were complementary to the sequences at the ends of the templates to make the linear templates into a loop and then use Splint to connect the gaps to obtain a complete template for amplification.

Synthesis of the DNA device TM

Concentrations of purified oligonucleotides (L, S1-1, S1-2, S1-3, S1, S2, S3, 1-4, 1-5, and 1-6) were determined by measuring UV absorbance at 260 nm. The purified DNA strands were mixed at the same molar ratio in a 1×TAE/Mg^{2+} buffer (40 mM Tris, 20 mM acetic acid, 2 mM EDTA, and 12.5 mM magnesium acetate, pH = 8.3) with a concentration of 1 μM. The aforementioned assembly solutions were kept at 95 °C for 3-5 min and then cooled down from 95 to 25 °C over 24 h.

Preparation of the DNA nanozyme

The preparation of the nano flower was based on isothermal rolling circle amplification (RCA) reaction as described.[1] Briefly, 2 μM 5′ phosphorylated DNA templates were mixed with an equivalent amount of primer and annealed in 1 × T4 DNA ligation buffer (50 mM Tris-HCl, 10 mM $MgCl_2$, 10 mM dithiothreitol (DTT), and 1 mM adenosine triphosphate (ATP), pH 7.5). This step was followed by the introduction of T4 DNA ligase (10 U/μL) and incubation at 16 °C for 12 h. Subsequently, the resultant circular DNA templates (0.3 μM) were incubated with dNTPs (1 mM) and phi29 DNA polymerase (1 U/μL) in the appropriate reaction buffer (50 mM Tris-HCl, 10 mM $MgCl_2$, 66 mM KCl, 0.04% (v/v) Tween 20, 4 mM DTT, pH 7.5) at 30 °C for 18 h.

After the reaction, the drug loading procedure for preparing NGH@AD was conducted through directly adding the component Hemin (3 eq to G4 DNAzyme) and Alexidine (AD). To optimize the loading efficacy of the therapeutic drug, different concentrations of AD (0.5 to 2.0 mg/mL) was added with different culture time (1.0 to 3.0 h). After incubation, the drug loading efficacy (DLE) was measured by high-performance liquid chromatography (HPLC, the mobile phase used a binary 10-min two-step gradient with solvent A containing ddH_2O with 0.1% trifluoroacetic acid and solvent B containing Optima grade acetonitrile with 0.1% trifluoroacetic acid. The gradient was 95:5–70:30 over 5 min and then 70:30–5:95 over the second 5 min.) and calculated according to the following equations:

$$\text{DLE}\ \% = \frac{\text{AD added} - \text{free AD}}{\text{AD added}} \times 100\%$$

For the fabrication of the final DNA nanozyme with DNA device modification, namely TMGH@AD, 10 nM of TM was mixed with NGH@AD at room temperature for 9 h. After the incubation, the TMGH@AD was washed with PBS and stored at 4 °C before use.

Characterization of TMGH@AD

TMGH@AD (1μg/mL in ddH_2O) was deposited onto a copper grid. After deposition for 0.5 h, the grid was dried overnight and sputter-coated with gold before observation. The images were collected and processed using Hitachi SU8220 Scanning Electron Microscope.

The prepared TMGH@AD (1μg/mL in ddH_2O)was deposited onto freshly cleaved mica and deposited for 10 min. After deposition, the surface of the mica was washed with 1 mL ddH_2O and air dry. The prepared samples were imaged with a MultiMode 8 AFM (Bruker) under ScanAsyst-Air mode. Dynamic light scattering (DLS) analysis of NGH@AD and TMGH@AD was performed on a Malvern Zetasizer Nano-ZS (Malvern Instruments, U.K.). The DLS analysis was dispersed in PBS, and the test was conducted at 25 °C.

Measurement of peroxidase-like Activity

The peroxidase-like activity of the DNA nanozyme in this study was verified using TMB as the substrate. The oxidation of TMB (200 μM) using H_2O_2 (200 μM) catalyzed by Hemin, DNAzyme, DNA nanoflower, and TMGH@AD in NaAc buffer in the presence of GSH was studied. The concentration of each component was based on 20 μM Hemin. The absorbance of oxidized TMB at 650 nm was monitored to assess the catalytic performance of the CDT agents.

Stability analysis

The TMGH@AD were incubated in DMEM with 10% FBS for 0 to 24 h at 37 °C. The concentration of the TMGH@AD was 10 μg/mL. After incubation, the samples were characterized by DLS to measure the particle size and distribution at 25 °C and 0.8% agarose gel electrophoresis to verify the aggregation and degradation.

***In vitro* BBB model**

For the *in vitro* BBB model, bEnd.3 cells were cultured for 72 h in gelatin-coated 0.4 μm pore size transwell plates until confluent. NGH@AD and TMGH@AD were labeled with Cy5 by using Cy5-modified DNA strand. Cy5-labeled ssDNA 1-6 (for Cy5-modified NGH@AD) and MT motif containing Cy5-labeled ssDNA 1-6 (for Cy5-modified TMGH@AD) were introduced for constructing the DNA nanostructure. The transcytosis of Cy5-labeled NGH@AD or aptamers-modified TMGH@AD was measured from the basal chamber using the IVIS imaging system at 12 h after the trans-endothelial electrical resistance of the model reached 100 $\Omega\cdot cm^2$. Subsequently, 1 μg/mL Cy5-labeled NGH@AD or Cy5-labeled TMGH@AD were added and after different time of treatment, fluorescence signals are collected for analyzing the effectiveness of crossing the BBB.

Cellular internalization and distribution analysis

The cell internalization and intracellular distribution of TMGH@AD were investigated with a confocal laser scanning microscope (LSM 980 CLSM, Carl Zeiss, Jena, Germany). 1×10^6 GL261 cells were seeded in a confocal plate for 12 hours for cell adhesion. The culture medium was replaced with 1 mL fresh medium containing Cy5-labeled TMGH@AD (1 μg/mL) and incubated for further 3 h at 37 °C. After incubation, the cells were washed with cold PBS for 3 times and stained with dyes for organelle imaging. Mitochondria extracted from Cy5-labeled NGH@AD or TMGH@AD using Qproteome Mitochondria Isolation Kit (Qiagen 37612) was imaged by fluorescence microscope.

3D cell spheroids were prepared by adding 1.5% agarose gel (200 μL) on the surface of the T75 flask. After 72 h of culture, GL261 spheroids were collected by centrifugation (100 g, 3 min) and added with 1 μg/mL Cy5-labeled TMGH@AD in medium. After 3 h of incubation, spheroids were washed with cold PBS for 3 times and stained with dyes for organelle

imaging.

AD accumulation analysis

GL261 cells were treated with Mixture (Nano flower + TM + Hemin + AD), NGH@AD, or TMGH@AD for 0 to 12 h. The drug concentration was based on 10 μg/mL AD. After treatment, cells were washed with cold PBS for 3 times and lysed by ultrasound. Then, the lysate supernatant was collected and filtered through a 0.22 μm syringe filter to remove particulate matter prior to HPLC analysis.

Cellular cytotoxicity assay

GL261 cells were seeded in 96-well plate with 4×10^3 cells per well and cultured overnight for cell adhesion. After that, the medium was replaced by 100 μL of fresh medium containing 100 μM H_2O_2 and PBS, Nano flower, AD, Mixture (Nano flower+ TM + Hemin + AD), NGH@AD, TMGH@AD. The drug concentration was based on 0 to 1000 ng/mL AD. The cells were further incubated for 48 h at 37 °C. Then the medium was replaced by DMEM and Cell Counting Kit-8 (CCK8) solution was added and incubated for 0.5 h at 37 °C. After the incubation, the absorbance at 450 nm was measured using a spectrophotometer.

Determination of Intracellular ROS

Intracellular ROS generation was assessed by using a DCFH-DA detector according to the manufacturer's instructions. Cells were seeded in confocal plates at a density of 1×10^5 cells per well. After cell adhesion, cells were treated with Mixture (Nano flower+ TM + Hemin + AD), NGH@AD, TMGH@AD for 12 h. The drug concentration was based on 1 μg/mL AD. For 3D spheroids, the drug concentration was based on 10 μg/mL AD. After incubation, the ROS probe DCFH-DA was added and the cells or spheroids were transferred for 30 min culture at 37 °C in the dark. Then, cells were washed thrice with PBS to remove extracellular DCFH-DA. Fluorescence imaging was performed using an inverted fluorescence microscope with an excitation wavelength of 488 nm and emission wavelength of 525 nm.

Mitochondrial membrane potential (MMP) detection.

After treatments with PBS, Mixture (Nano flower+ TM + Hemin + AD), NGH@AD, or TMGH@AD for 12 h (the drug concentration was based on 1 μg/mL AD), the GL261 cells were incubated with a JC-1 staining solution for 20 min at 37 °C and washed off with PBS, and the images were obtained by a fluorescent inverted microscope Confocal Imaging System (Leica Microsystems) with randomly selected fields of vision, respectively and analyzed by ImageJ software.

Transmission Electron Microscopy (TEM) Analysis.

GL261 cells after treatments were prefixed with 2% glutaraldehyde and fixed in 1% osmium tetroxide. Next, the samples were dehydrated in ethanol containing 3% uranyl acetate, embedded in epoxy resin and propylene oxide overnight, and polymerized into 70 nm thick slices and stained with lead citrate, using H-7800 transmission electron microscopy (Hitachi).

Intracellular GSH and disulfide levels

GL261 cells after treatments were washed with cold PBS for 3 times. The intracellular GSH levels and disulfide level were analyzed using GSH and GSSG assay kits following the instructions, and the disulfide level were analyzed using GSH and GSSG assay kits without

the protein clearance process.

Intracellular $NADP^+$/NADPH ratio

$NADP^+$/NADPH assay kit with WST-8 was used to evaluate the intracellular $NADP^+$/NADPH ratio. GL261 cells after treatments were washed with cold PBS for 3 times. Then, $NADP^+$/NADPH extract was added to the cell lysate and gently mixed to promote cell lysis. This was followed by centrifugation at 12,000 g, 4 ºC for 5 min and the supernatants were taken as samples to be tested. The total amount of $NADP^+$ and NADPH was detected using a microplate reader (SpectraMax) at 450 nm (peak absorbance). Then, the sample to be tested at the beginning was heated in a water bath for 0.5 h to remove $NADP^+$ as a new sample to be tested, and the amount of NADPH was measured using the same method above. Finally, the protein concentration was measured using the BCA protein assay kit, and $NADP^+$/NADPH ratio was calculated.

Animal Experiment Ethics

C57 mice (6-8 weeks old) were purchased from Vital River Laboratory Animal Technology Co. Ltd. All animals received care in compliance with the guidelines outlined in the Guide for the Care and Use of Laboratory Animals. The procedures were approved by the Institutional Animal Care and Use Committee of Hainan Medical University with ethics approval (HYLL-2024-253). All surgical procedures followed the standard guidelines.

***In vivo* therapeutic effect**

To establish a GL261 glioma mouse model, 6-week-old C57BL/6 mice are positioned in a stereotactic frame. After positioning, 5 μL of the GL261-LUC cell suspension was slowly injected through stereotactic microinjection. Postoperatively, the tumor growth is confirmed through bioluminescence imaging. Mice bearing GL261-LUC gliomas were randomly assigned to four groups (PBS, Mixture, NGH@AD, and TMGH@AD) and received vein injection with 50 μL drugs. The drug concentration was based on 2 mg AD/kg body weight. Tumor progression was monitored by measuring bioluminescence at various time points following injection. Additionally, the survival rate for each group was documented with body weight measured every other day.

Staining of tissue sections

After being fixed with 4% paraformaldehyde, mouse organs and tumors were embedded in paraffin blocks, sectioned at 5 μm thickness using a microtome, and stained with hematoxylin and eosin staining (H&E) for microscopic examination. Terminal deoxynucleotidyl transferase dUTPbiotin nick ends (TUNEL) fluorescence staining was utilized to visualize apoptosis in tumor cells. Fluorescence was analyzed under a fluorescence microscope to examine DAPI/FITC-12-dUTP-stained tissue slices immediately to avoid fluorescence quenching.

For immunofluorescence analysis, the brain tissues harvested after the treatment were cross-sectioned to 5 μm thick slices and the slices were mounted on slides for staining. The immunostaining analysis of PMPTI, ROS, and Ki67 were conducted by a standard protocol according to the manufacturer's instructions.

Statistical analysis

Data represent the mean ± s.d. from indicated independent replicates. Statistical analysis

was conducted using GraphPad Prism. For comparisons between two groups, means were compared using the one-way analysis of variance (ANOVA) with the Tukey post hoc test.

A value of $P < 0.05$ was considered statistically significant.

3. Supporting figures

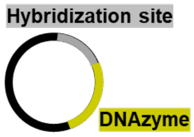

TTTGTGCTACTCCAGTTCTTTTTGGGTAGG
GCGGGTTGGGTT

Figure S1. Illustration and sequence of the template.

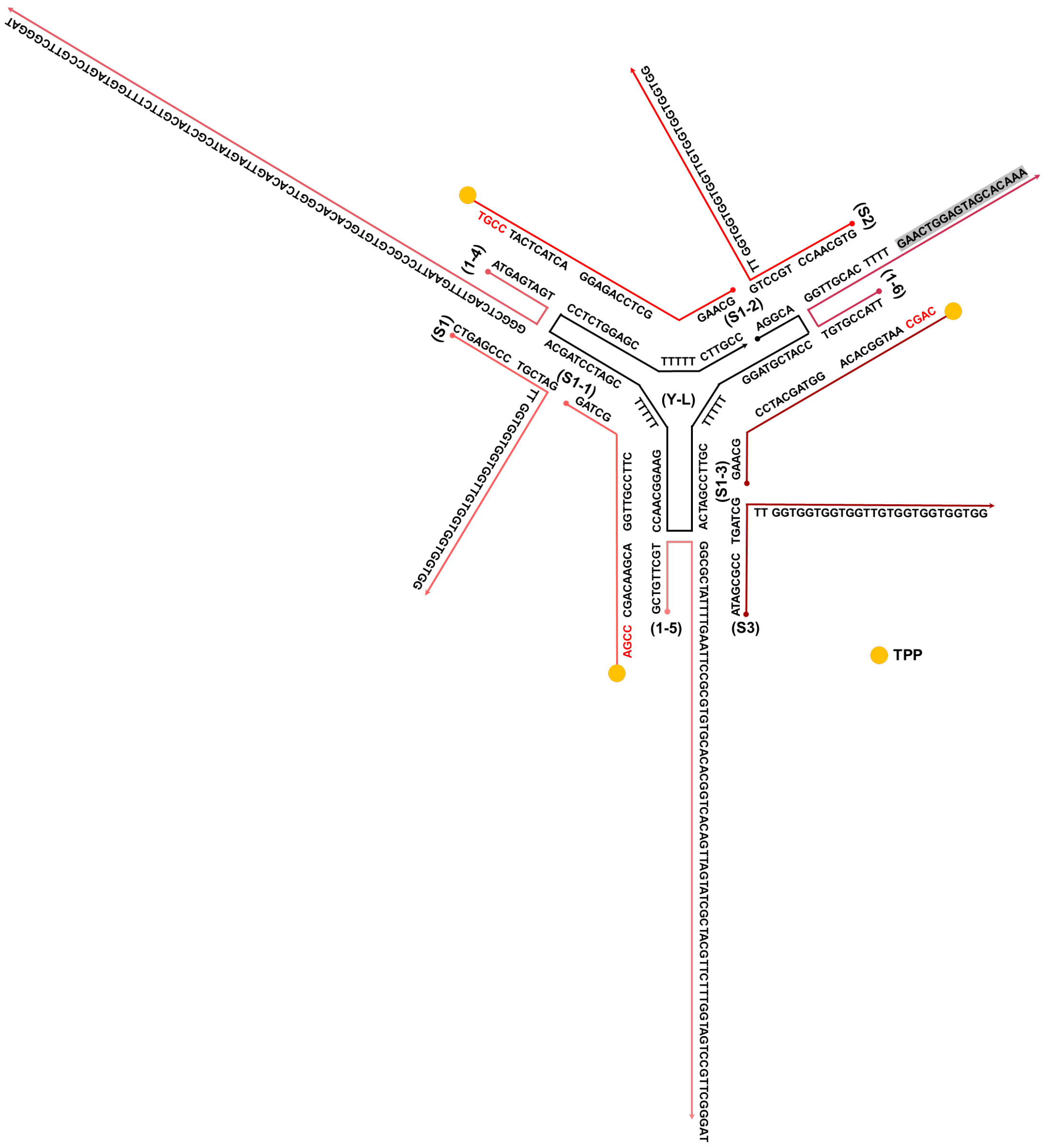

Figure S2. Illustration of the construction of the DNA device TM. TPP: triphenylphosphine modification.

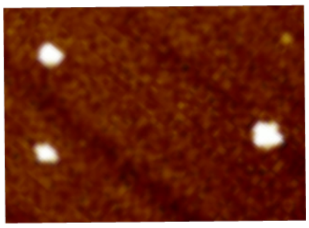

Figure S3. Atomic force microscope imaging of the DNA Nano flower under ScanAsyst-Air mode.

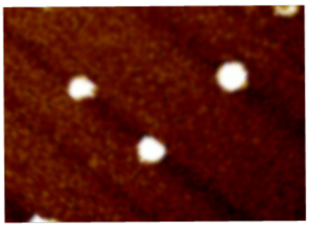

Figure S4. Atomic force microscope imaging of the NGH@AD under ScanAsyst-Air mode.

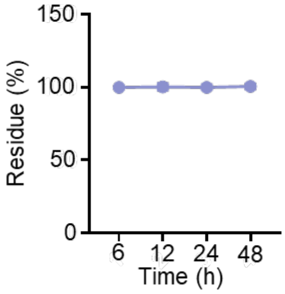

Figure S5. Quantification of the 0.8 % agarose gel electrophoresis.

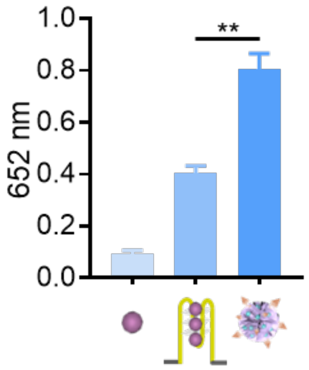

Figure S6. Absorbance at 652 nm for TMB oxidation. (n=3). Statistical significance was calculated by one-way ANOVA with the Tukey post hoc test ($p > 0.05$, *$p < 0.05$, **$p < 0.01$, ***$p < 0.001$, and ****$p < 0.0001$).

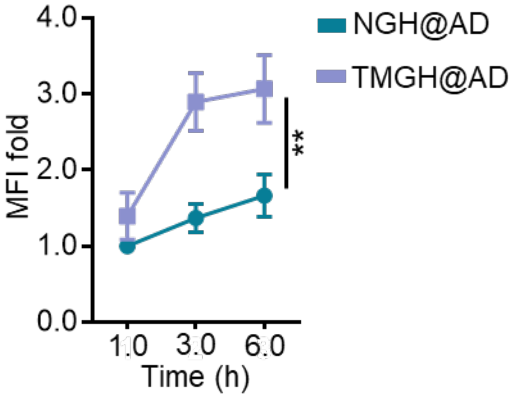

Figure S7. Time-dependent cellular uptake measured by Flow cytometry. The data are presented as mean ± SEM (n=3). Statistical significance was calculated by one-way ANOVA with the Tukey post hoc test (**p < 0.01).

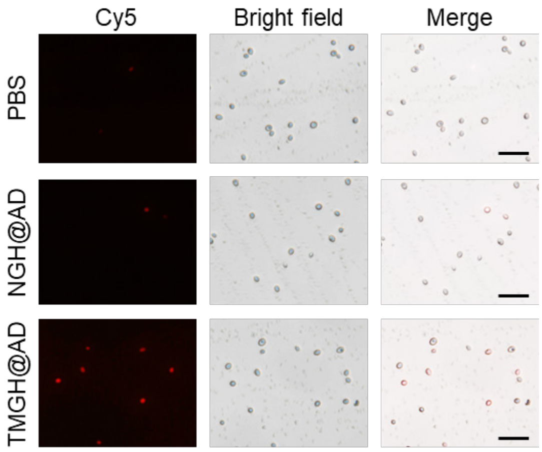

Figure S8. Fluorescence imaging of the mitochondria directly extracted from Cy5-labeled NGH@AD or TMGH@AD-treated cells. Scale bar: 5 μm.

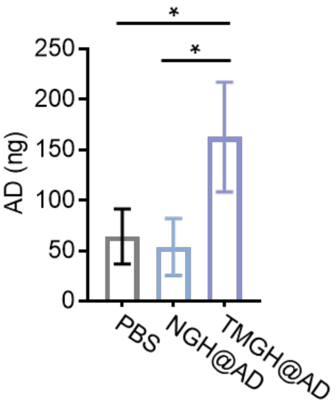

Figure S9. AD accumulation in mitochondria measured by HPLC. (n=3). Statistical significance was calculated by one-way ANOVA with the Tukey post hoc test (*$p < 0.05$).

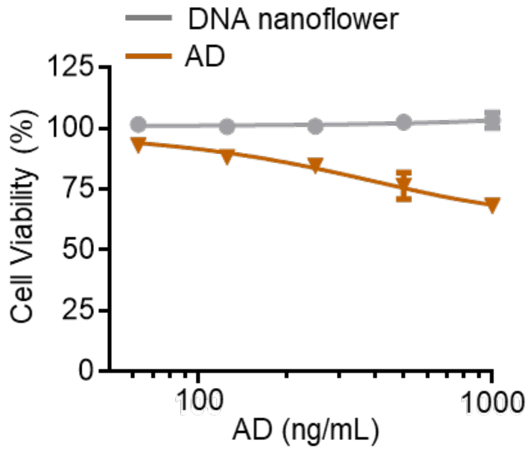

Figure S10. Cell viability analysis of GL261 cells treated with Nano flower or free AD. The drug concentration was based on AD (0 to 1000 ng/mL). The data are presented as mean ± SEM (n=3).

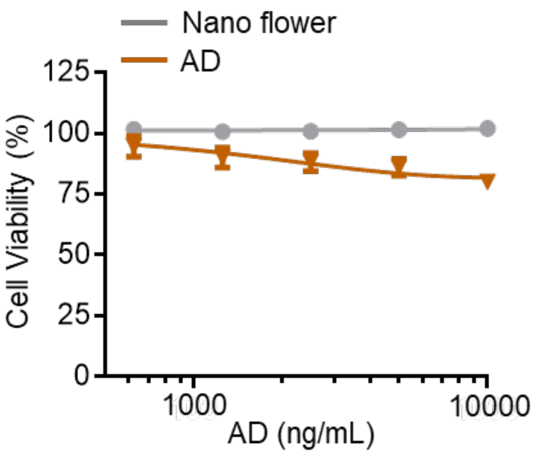

Figure S11. Cell viability analysis of GL261 spheroids treated with Nano flower or free AD. The drug concentration was based on AD (0 to 10000 ng/mL). The data are presented as mean ± SEM (n=3).

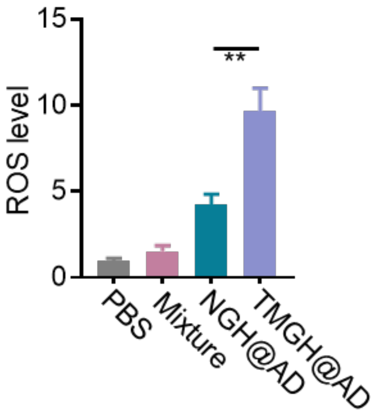

Figure S12. ROS level in GL261 cells. (n=3). Statistical significance was calculated by one-way ANOVA with the Tukey post hoc test (**p < 0.01).

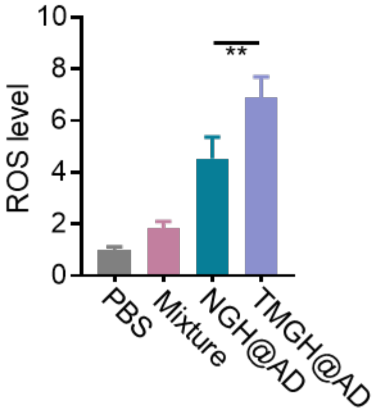

Figure S13. ROS level in GL261 spheroids after different treatments. (n=3). Statistical significance was calculated by one-way ANOVA with the Tukey post hoc test (**$p < 0.01$).

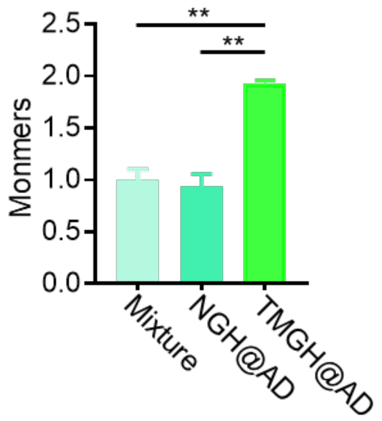

Figure S14. Monomer level measured by JC-1 staining. (n=3). Statistical significance was calculated by one-way ANOVA with the Tukey post hoc test (**$p < 0.01$).

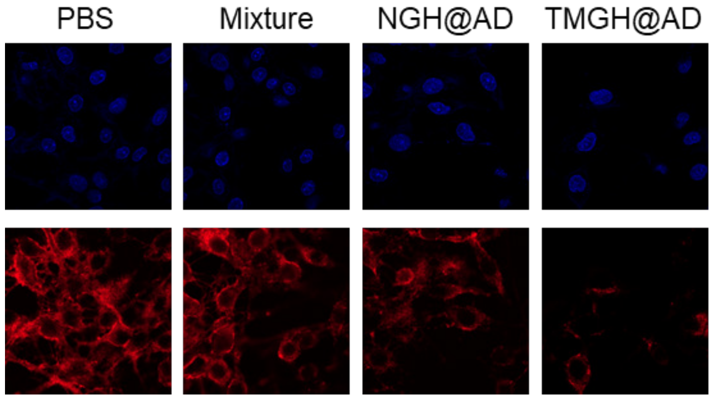

Figure S15. CLSM images of PMPT1 in GL261 cells after different treatments.

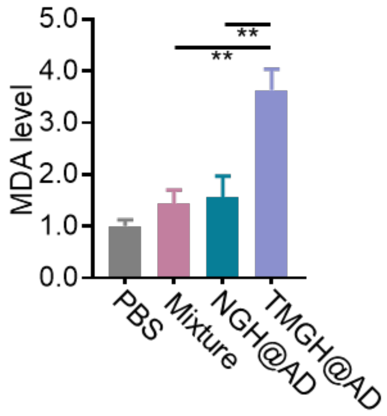

Figure S16. Intracellular MDA level in GL261 cells after different treatments. (n=3). Statistical significance was calculated by one-way ANOVA with the Tukey post hoc test (** $p < 0.01$).

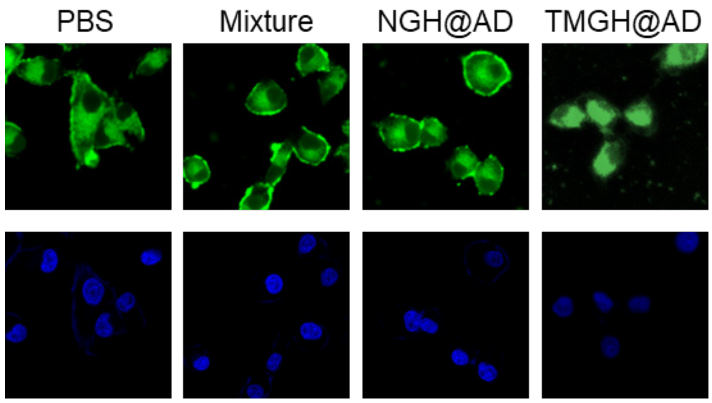

Figure S17. CLSM images of F-actin in GL261 cells.

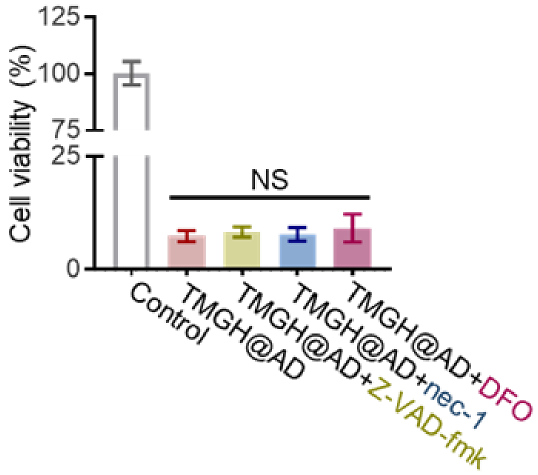

Figure S18. Cell viability of GL261 cells post-treatment with TMGH@AG plus 25 nM Z-VAD-fmk, nec-1, or DFO. The DNA nanozyme concentration was based on 1 µg/mL AD. The data are presented as mean ± SEM (n=3). Statistical significance was calculated by one-way analysis of variance (ANOVA) with the Tukey post hoc test.

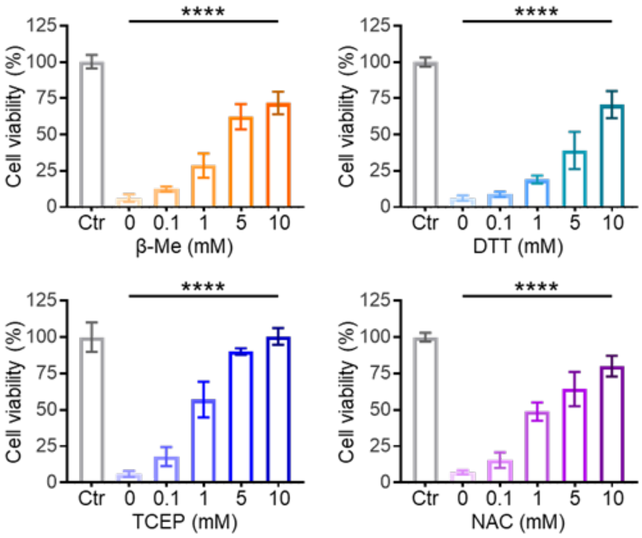

Figure S19. Cell viability of GL261 under the treatment of TMGH@AD plus different concentrations of reducing agent. (n=3). Statistical significance was calculated by one-way ANOVA with the Tukey post hoc test (**** $p < 0.0001$).

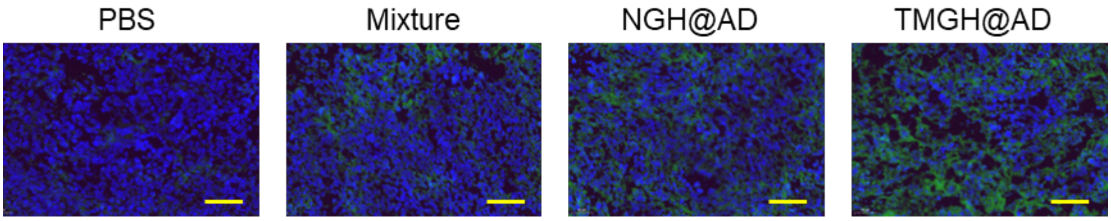

Figure S20. TUNEL staining in different groups. Scale bar: 40 μm.

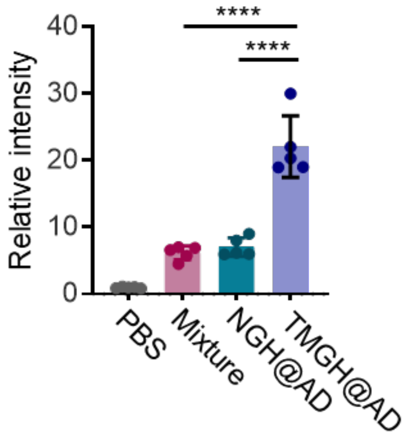

Figure S21. Relative intensity of the TUNEL signal in glioma tissue. (n=3). Statistical significance was calculated by one-way ANOVA with the Tukey post hoc test (**** $p < 0.0001$).

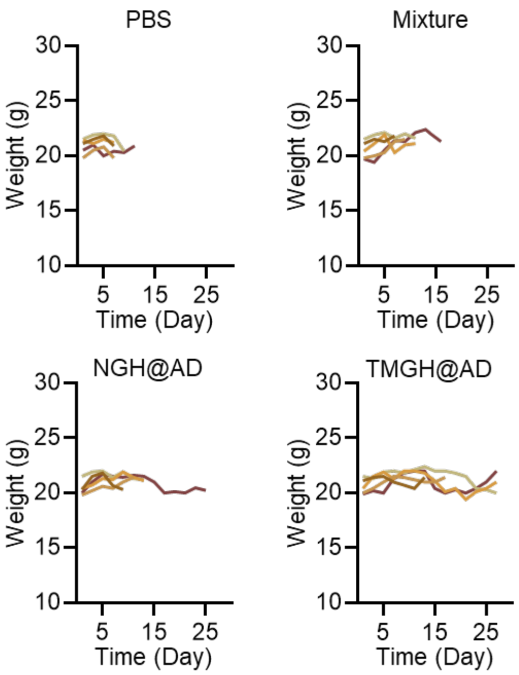

Figure S22. Body weight curves of the mice during the treatment.

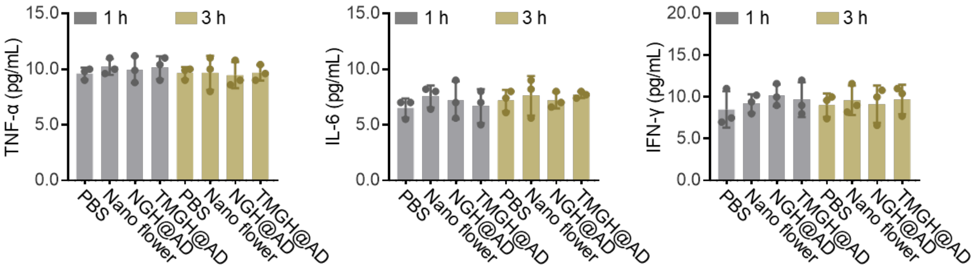

Figure S23. Concentration of cytokines in blood serum at 1 and 3 h postinjection. The data are presented as mean ± SEM (n=3).

4. Supporting tables

Table S1. DNA sequences in this study.

Name	Sequence (5'-3')
**Template**	Phosphate-TTTGTGCTACTCCAGTTCTTTTTGGGTAGGGCGGGTTGG GTT
**L**	AGGCACCATCGTAGGTTTTTCGTTCCGATCACCAACGGAAGTTTTT CGATCCTAGCACCTCTGGAGCTTTTTCTTGCC
**S1-1**	GATCGCTTCCGTTGGACGAACAGCCCGA-TPP
**S1-2**	AAGCAAG GCTCCAGAGGACTACTCATCCGT-TPP
**S1-3**	GAACG CCTACGATGGACACGGTAACGAC-TPP
**S1**	CTGAGCCCTGCTAGTTGGTGGTGGTGGTTGTGGTGGTGGTGG
**S2**	GTGCAACCTGCCTGTTGGTGGTGGTGGTTGTGGTGGTGGTGG
S3	ATAGCGCCTGATCGTTGGTGGTGGTGGTTGTGGTGGTGGTGG
**1-4**	ATGAGTAGTGGGCTCAGTTTGAATTCCGCGTGTGCACACGGTCAC AGTTAGTATCGCTACGTTCTTTGGTAGTCCGTTCGGGAT
**1-5**	GCTGTTCGTGGCGCTATTTTGAATTCCGCGTGTGCACACGGTCACA GTTAGTATCGCTACGTTCTTTGGTAGTCCGTTCGGGAT
**1-6**	TTACCGTGTGGTTGCACTTTTGAACTGGAGTAGCACAAA

5. References

[1] a)C. Yao, R. Zhang, J. Tang, D. Yang, *Nature Protocols* **2021**, 16, 5460; b)R. Tian, Y. Wang, H. Wang, Z. Wang, Y. Chen, J. Fan, B. Li, Q. Jiang, B. Ding, *CCS Chemistry* **2025**, 7, 1745.

[2] a)H. Shoji, T. Miyakawa, *Neuropsychopharmacology Reports* **2019**, 39, 100; b)B. Li, Q. Zhang, J. Cheng, Y. Feng, L. Jiang, X. Zhao, Y. Lv, K. Yang, J. Shi, W. Wei, P. Guo, J. Wang, M. Cao, W. Ding, J. Wang, D. Su, Y. Zhou, R. Gao, *ACS Nano* **2024**, 18, 21061.

[3] J. Wang, Y. Wang, X. Xiaohalati, Q. Su, J. Liu, B. Cai, W. Yang, Z. Wang, L. Wang, *Advanced Science* **2023**, 10, 2206854.
